# Excessive salt intake accelerates the progression of cerebral small vessel disease in older adults

**DOI:** 10.1186/s12877-023-03877-3

**Published:** 2023-05-02

**Authors:** Di Liu, Qin Zhang, Shasha Xing, Fang Wei, Ke Li, Yingxin Zhao, Hua Zhang, Gary Gong, Yuqi Guo, Zhendong Liu

**Affiliations:** 1 Department of Cardiology, Shandong Provincial Hospital Affiliated to Shandong First Medical University, No. 324, Jingwuweiqi Road, Jinan, Shandong 250021 China; 2 School of Basic Medicine, Shandong First Medical University & Shandong Academy of Medical Sciences, Jinan, Shandong 250117 China; 3 Department of Geriatrics, the Third Hospital of Lixia District, Jinan, Shandong 250100 China; 4 Department of Cardiology, Jinan Central Hospital Affiliated to Shandong First Medical University, Jinan, Shandong 250013 China; 5 The Russel H. Morgan Department of Radiology and Radiological Sciences, the Johns Hopkins University School of Medicine, Baltimore, MD 21287 USA

**Keywords:** Aging, Cerebral small vessel disease, Harmful effect, Progression, Salt intake

## Abstract

**Background:**

It is unclear whether excessive salt intake accelerates the progression of cerebral small vessel disease (CSVD). The major objective of this study was to investigate the harmful effect of excessive salt intake on the progression of CSVD in older individuals.

**Methods:**

Between May 2007 and November 2010, 423 community-dwelling individuals aged 60 years and older were recruited from the Shandong area, China. Salt intake was estimated using 24-hour urine collection for 7 consecutive days at baseline. Participants were classified into low, mild, moderate and high groups according to the salt intake estimation. CSVD including white matter hyperintensities (WMHs), lacunes, microbleeds and an enlarged perivascular space (EPVS) were determined using brain magnetic resonance imaging.

**Results:**

During an average of five years of follow-up, the WMH volume and WMH-to-intracranial ratio were increased in the four groups. However, the increasing trends in the WMH volume and WMH-to-intracranial ratio were significantly faster in the higher salt intake groups compared with the lower salt intake groups (*P*_adjusted_ < 0.001). The cumulative hazard ratios of new-incident WMHs (defined as those with Fazekas scale scores ≥ 2), new-incident lacunes, microbleeds or an EPVS, as well as composites of CSVD, were respectively 2.47, 2.50, 3.33, 2.70 and 2.89 for the mild group; 3.72, 3.74, 4.66, 4.01 and 4.49 for the moderate group; and 7.39, 5.82, 7.00, 6.40 and 6.61 for the high group, compared with the low group after adjustment for confounders (*P*_adjusted_ < 0.001). The risk of new-incident WMHs, lacunes, microbleeds or an EPVS, and composites of CSVD was significantly increased with each 1-standard-deviation increment in salt intake (*P*_adjusted_ < 0.001).

**Conclusion:**

Our data indicates that excessive salt intake is an important and independent contributor to the progression of CVSD in older adults.

## Introduction

Cerebral small vessel disease (CSVD) is one of the most common, chronic and age-related vascular diseases with a cluster of neuroimaging manifestations including white matter lesions (WMLs), lacunes, microbleeds and an enlarged perivascular space (EPVS) [1–3]. CSVD has been demonstrated to contribute to more than 25% of strokes and 45% of dementia cases [2–5]. With increasing life expectancy, CSVD poses a higher burden on individuals and societal healthcare worldwide [1, 3, 5].

It has been demonstrated that the etiology of CSVD is varied. Known risk factors include aging, hypertension [6], diabetes [7] and dyslipidemia [8], as well as unhealthy life styles and dietary patterns [9–11]. Excessive salt intake is one of the most common unhealthy dietary patterns. Salt (sodium chloride, NaCl), a major food additive, is an essential nutrient for the maintenance of normal physiological reactions. However, excessive salt intake is well accepted as an important risk factor for damage to multiple organs involving hypertension, atherosclerosis, heart failure and stroke [12, 13]. Evidence has shown that approximately 30% of diet-related deaths are caused by excessive salt intake [14, 15]. However, the association between excessive salt intake and the risk of CSVD progression is still incompletely understood.

In this study, we assessed salt intake estimated by 24-hour urine collection for 7 consecutive days in 423 individuals aged 60 years or older, and then followed them for an average of 5 years to shed light on the association between salt intake and CSVD progression.

## Materials and methods

### Study population and design

Between May 2007 and November 2010, 423 older adults aged 67.48 ± 5.48 years (range, 60–82 years) were enrolled from the community dwelling in the Shandong area, China. Among them, 251 (59.3%) were female, and 172 (40.7%) were male. The exclusion criteria were as follows: history of stroke, history of transient ischemic attack, Alzheimer’s disease, Parkinson’s disease, psychosis, congestive heart failure, myocardial infarction, atrial fibrillation, dialysis treatment, chronic and acute renal diseases, severe liver diseases, drug and alcohol abuse and chronic or acute liver diseases. Also excluded were people with contraindications to magnetic resonance imaging (MRI), those with uncompleted 24-hour urine collection for 7 consecutive days, and those unwilling to provide informed consent.

The design of this study complied with the Declaration of Helsinki and was approved by the Research Ethics Committee of the Institute of Basic Medicine, Shandong Academy of Medical Sciences, China. Written informed consent was obtained from each participant.

### Estimation of salt intake

In this study, salt intake was estimated using 24-hour urine collection for 7 consecutive days. This method is widely used to estimate daily salt intake and has been endorsed by the World Health Organization. Participants were asked to maintain their usual daily dietary patterns and were instructed to collect 24-hour urine for a 7-day consecutive period. Na + concentration (unit, mmol/L) of the urine sample was measured using a DSI-905 electrolyte analyzer (Shanghai Xunda Medical Instrument Co., Ltd., Shanghai, China). Salt intake per day was calculated using the following formula:

$$\begin{gathered}salt - intake\,(g/day) = [concentration\,of\,N{a^ + }(mmol/L) \times \hfill \\\,\,\,\,\,\,\,\,\,\,\,\,\,\,\,\,\,\,\,\,\,\,\,\,\,\,\,\,\,\,\,\,\,\,\,\,\,\,\,\,\,\,\,\,\,\,\,\,\,\,urine\,volume\,(L/day)] \div17\,(mmol/g) \hfill \\\end{gathered}.$$

Urine creatinine excretion was measured and used to correct the quality and quantity of the urine sample. The urine samples were collected in the spring or autumn to minimize sweating which may bias the results [16]. We used the spot urine with Tanaka equation to determine the changes of salt intake styles across the follow-up period [17].

### Brain MRI

CSVD assessment was conducted using 3.0-Tesla scanner (Signa Horizon LX, GE Medical Systems, Pittsburgh, PA, USA; Siemens Medical, Erlangen, Germany) with a uniform protocol as described elsewhere [4, 16]. The scan consisted of T1-weighted 3D magnetization-prepared rapid-gradient echoes (repetition time [TR]/echo time [TE] = 1900/3, slice thickness = 1 mm, no gap), T2-weighted 3D fast-spin echoes (TR/TE = 3000/98, slice thickness = 3 mm, no gap), T2*-weighted gradient-echo type echoplanar sequences (TR/TE = 600/16, slice thickness = 3 mm, no gap), and fluid-attenuated inversion recovery sequences (FLAIR; TR/TE = 5000/335, slice thickness = 2 mm, no gap). White matter hyperintensity (WMH) volumes were computed automatically from periventricular and subcortical segmentation on FLAIR axial images using the BET and FAST tools from the FSL 4.19 software package (http://www.fmrib.ox.ac.uk/fsl). Total intracranial volume (ICV) was used to correct the WMH volume. WMH was also visually rated as none, punctuate cap, early confluent or confluent (scored from 0 to 3, respectively) according to the Fazekas rating scale [4, 18].

Cerebral lacunes, microbleeds and an EPVS were also determined in this study according to the diagnostic criteria defined in STRIVE v1 [19]. Lacunes were defined as cavities of cerebrospinal fluid-like signals with a diameter of 3–15 mm; microbleeds as round or oval, hypointense or homogeneous foci with a diameter of 2–10 mm that could be distinguished from calcifications, signal averaging from bone and sulcal vessels; and an EPVS as a visible fluid-filled space adjacent to cerebral vessels. Lacunes were assessed using a combination of T1, T2 and FLAIR images, microbleeds using T2* images and an EPVS using a combination of T2 and FLAIR images.

All scan images were transferred to an offline workstation and rated in a side-by-side fashion by experienced neuroradiologists who were blinded to the salt intake estimation and clinical data of participants. The inter-observer variability was 0.91 for WMH volume, 0.87 for Fazekas scale scores, 0.83 for lacunes and microbleeds and 0.82 for an EPVS after randomly rating 50 MRI scans.

### Follow-up

After the baseline visit, participants were visited annually to acquire demographic and clinical characteristics with the help of family physicians or nurses. Brain MRI scans and salt intake estimation of each participant were conducted at baseline (2007–2010), the first brain MRI and salt intake estimation follow-up (2010–2013), the second follow-up (2013–2015), and the third follow-up (2015–2018). The participants who changed the salt intake style during the follow-up period were excluded and removed from the study.

### Outcomes

The primary outcomes included the progression of CSVD load, WMLs, lacunes, microbleeds and an EPVS. The progression of CSVD load was defined as the composite of new-incident WMHs (defined as those with Fazekas scale scores ≥ 2), as well as new-incident lacunes, microbleeds and an EPVS. It was rated as 1 if one of the components occurred, and the total score ranged from 1 to 4. The progression of WMLs was assessed using changes in the WMH volume and WMH-to-ICV ratio as well as new-incident WMHs across the follow-up duration. The progression of lacunes, microbleeds and an EPVS was presented as the first occurrence of new-incident lacunes, microbleeds and an EPVS during the follow-up period. New-incident events were defined one or more new incidents of WMH with Fazekas scale scores ≥ 2, or new incidents of lacunes, microbleeds or an EPVS [18]. The second outcome was stroke, which was defined as the first occurrence of a neurological deficit lasting longer more than 24 h with relevant findings of cerebral hemorrhage or infarction in MRI or computed tomography.

### Statistical analysis

Depending on the salt intake estimation, participants were classified into low (≤ 6.00 g/day of salt intake), mild (6.01–9.00 g/day of salt intake), moderate (9.01–12.00 g/day of salt intake), and high (> 12.00 g/day of salt intake) groups depending on the Dietary Guidelines for Chinese Residents [17, 20]. Characteristics of participants in different groups are presented as means ± standard deviation (SD) or medians with interquartile range for measurement data depending on the normality, and frequency with percentages for enumeration data. The Kolmogorov-Smirnov test was used to assess the normality of measurement data. The changes in the WMH volume and WMH-to-ICV ratio were defined as the differences between the value assessed at follow-up visits minus the value assessed at baseline visit. The correlations between salt intake and the incidence of CSVD were assessed using Spearman’s correlation coefficients. The differences in measurement data among groups were assessed using one-way analysis of variance with Bonferroni post-hoc tests, or Kruskal-Wallis tests with Wilcoxon rank-sum tests. The Kruskal-Wallis test with Wilcoxon rank-sum test was also used to detected the differences in new CSVD load among groups. The chi-square test was used to assess differences in enumeration data. A linear mixed model was used to determine the differences in changes in WMH volumes and WMH-to-ICV ratios during the follow-up period among groups. Differences in the cumulative risk for progression of WMLs, lacunes, microbleeds and an EPVS among groups were estimated using Kaplan-Meier analyses with log-rank tests. The Cox proportional hazards model was used to determine the hazard ratio (HR) with 95% confidence interval (CI). The adjustments for models included age, sex, alcohol consumption, smoking, history of diseases and medications, baseline body mass index, baseline systolic and diastolic blood pressure, baseline lipids including total cholesterol, triglycerides, high-density lipoprotein-cholesterol, low-density lipoprotein-cholesterol, and baseline fasting plasma glucose. The baseline WMH volume was also included as a confounder in all models because WMH has been demonstrated to be closely associated with lacunes, microbleeds and an EPVS [21–23]. All statistical analyses were performed using SPSS v26.0 (SPSS Inc., Chicago, IL, USA), and a two-sided P value < 0.05 was considered statistically significant.

## Results

### Baseline characteristics

**Fig. 1:**
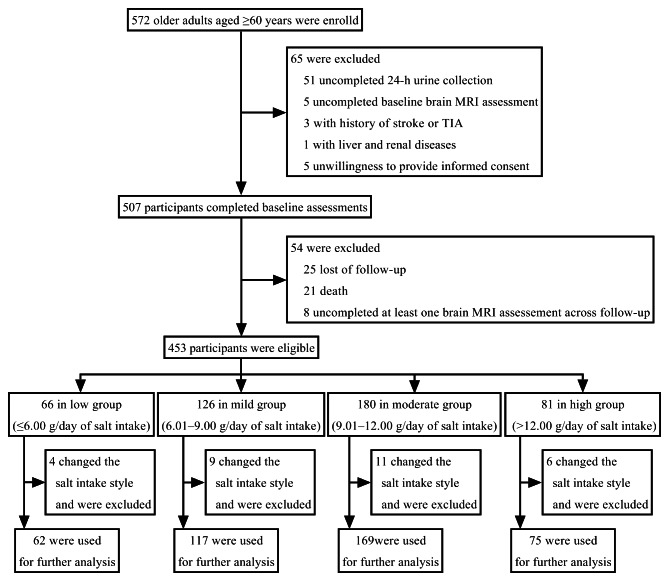
Study protocol flowchart. TIA, transient ischemic attack

The flowchart of this study is shown in Fig. 1. The range of the salt intake was from 4.40 to 14.91 g/day. The details of the baseline demographic and clinical characteristics, as well as the brain MRI assessment at baseline are summarized in Table 1. Compared with the low group, the WMH volume and WMH-to-ICV ratio were significantly higher in the mild, moderate and high groups (all *P* < 0.05). The WMH-to-ICV ratio in the high group was higher than in the mild group (*P* < 0.05).

**Table 1:** Demographics and clinical characteristics of participants

	Low group (*n* = 62)	Mild group (*n* = 117)	Moderate group (*n* = 169)	High group (*n* = 75)	*P* value
Clinical parameters					
Age (years)	66.60 ± 4.83	68.00 ± 6.11	67.29 ± 5.14	67.80 ± 5.69	0.374
Female (*n* [%])	35 (56.5)	69 (59.0)	97 (57.4)	50 (66.7)	0.541
Education (years)	6.87 ± 4.27	6.84 ± 4.51	6.74 ± 4.51	7.56 ± 4.36	0.599
Smoking (*n* [%])	15 (24.2)	28 (23.9)	50 (29.6)	23 (30.7)	0.607
Alcohol consumption (*n* [%])	25 (40.3)	37 (31.6)	53 (31.4)	30 (40.0)	0.377
Hypertension (*n* [%])	41 (66.1)	80 (68.4)	119 (70.4)	54 (72.0)	0.876
Antihypertensive medication (*n* [%])	36 (58.1)	66 (56.4)	86 (50.9)	31 (41.3)	0.150
Diabetes, *n* (%)	6 (9.7)	20 (17.1)	17 (10.1)	8 (10.7)	0.272
Anti-diabetes medication (*n* [%])	6 (9.7)	20 (17.1)	15 (8.9)	7 (9.3)	0.148
Dyslipidemia (*n* [%])	18 (29.0)	35 (29.9)	37 (21.9)	18 (24.0)	0.415
Anti-dyslipidemia medication (*n* [%])	4 (6.5)	9 (7.7)	11 (6.5)	3 (4.0)	0.788
Antiplatelet medication (*n* [%])	`10 (16.1)	11 (9.4)	29 (17.2)	11 (14.7)	0.312
Heart rate (bpm)	71.58 ± 9.45	69.833 ± 9.23	70.81 ± 7.73	72.55 ± 8.58	0.175
Body mass index (kg/m^2^)	24.59 ± 2.30	24.43 ± 2.40	24.33 ± 2.38	24.60 ± 2.55	0.818
Systolic blood pressure (mm Hg)	141.18 ± 14.72	145.13 ± 15.65	144.52 ± 15.42	145.60 ± 15.76	0.332
Diastolic blood pressure (mm Hg)	76.18 ± 8.16	76.53 ± 8.31	76.24 ± 8.47	77.28 ± 8.77	0.826
Biochemical parameters					
Total cholesterol (mmol/L)	4.59 ± 0.65	4.61 ± 0.72	4.66 ± 0.71	4.72 ± 0.63	0.651
Triglycerides (mmol/L)	1.61 ± 0.40	1.51 ± 0.47	1.61 ± 0.49	1.66 ± 0.48	0.134
HDL (mmol/L)	1.13 ± 0.30	1.15 ± 0.32	1.11 ± 0.32	1.10 ± 0.28	0.720
LDL-c (mmol/L)	2.73 ± 0.62	2.77 ± 0.62	2.81 ± 0.66	2.87 ± 0.58	0.584
FPG (mmol/L)	5.26 ± 0.94	5.30 ± 1.19	5.59 ± 1.31	5.46 ± 1.09	0.121
Brain MRI parameters					
WMH (ml)	3.75 (2.24 to 5.16)	4.44 (3.11 to 5.53)*	4.54 (3.26 to 5.83)*	4.69 (3.74 to 6.11)*	0.002
WMH-to-ICV ratio (%)	0.28 (0.18 to 0.41)	0.34 (0.23 to 0.42)*	0.35 (0.25 to 0.47)*	0.38 (0.30 to 0.49)*†	< 0.001
Fazekas scale ≥ 2 (*n* [%])	4 (6.5)	9 (7.7)	17 (10.0)	9 (12.0)	0.631
Lacunes (*n* [%])	3 (4.8)	10 (8.5)	19 (11.2)	8 (10.7)	0.491
Microbleeds (*n* [%])	2 (3.2)	7 (6.0)	13 (7.7)	7 (9.3)	0.508
EPVS (*n* [%])	4 (6.5)	9 (7.7)	17 (10.0)	11 (14.7)	0.331

*Notes*: Data are expressed as the mean ± standard deviation or numbers with percentages. HOH indicates home-measured orthostatic hypotension; HDL-c, high-density lipoprotein-cholesterol; LDL-c, low-density lipoprotein-cholesterol; FPG, fasting plasma glucose; WMH, white matter hyperintensities; ICV, intracranial; EPVS, enlarged perivascular space. *P < 0.05, compared with the low group; †*P* < 0.05, compared with the mild group

### Progression of WMLs

**Fig. 2:**
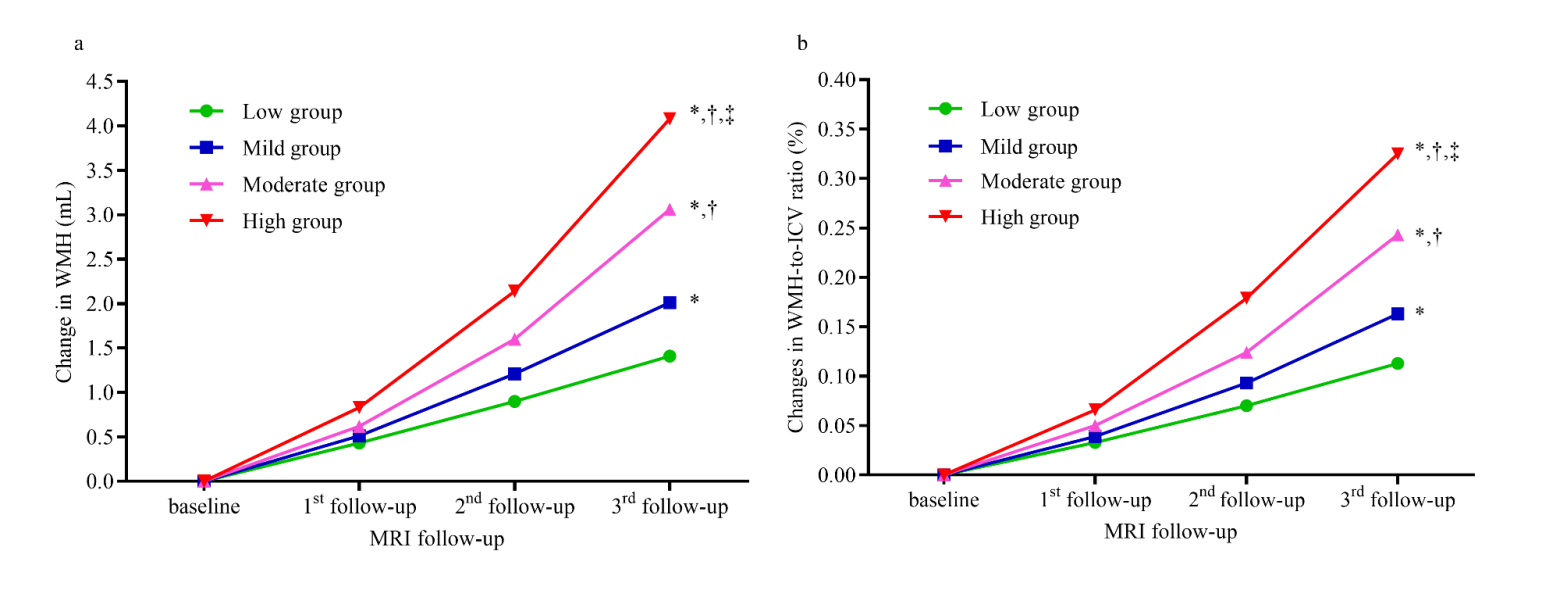
Differences in the progression of WMLs among groups. (a) Differences in changes in the WMH volume during the follow-up period among groups. (b) Differences in changes in the WMH-to-ICV ratio during the follow-up period among groups. WMH, white matter hyperintensity; ICV, intracranial volume. *P < 0.05, compared with the low group; †*P* < 0.05, compared with the mild group; ‡*P* < 0.05, compared with the moderate group

There were significant increases in the WMH volume and WMH-to-ICV ratio across the follow-up period in all four groups. The trend to increase was significantly different among the four groups even after adjustment for confounders including systolic and diastolic blood pressure, history of hypertension and antihypertensive medication, blood lipids, fasting plasma glucose, and baseline WMH volume (*P*_adjusted_ <0.001, Fig. 2). Furthermore, the increasing trend accelerated from the low to high group. There were significant differences in the WMH volume and WMH-to-ICV ratio between any two group after adjustment for confounders (all *P*_adjusted_ <0.05).

### Correlation between salt intake and the incidence of CSVD

During the study, 131 (4.4% per year) participants developed WMH with Fazekas scale scores ≥ 2, 159 (5.4% per year) participants developed lacunes, 148 (5.0% per year) developed microbleeds and 179 (6.1% per year) developed an EPVS. The new-incident WMH, lacunes, microbleeds, and EPVS, as well as the composite incident CSVD were significantly and positively correlated with salt intake (correlation coefficients were 0.202, 0.207, 0.188, 0.222, and 0.288, respectively; *P* < 0.001 for all).

### Differences in the progression of CSVD grouped by salt intake

**Fig. 3:**
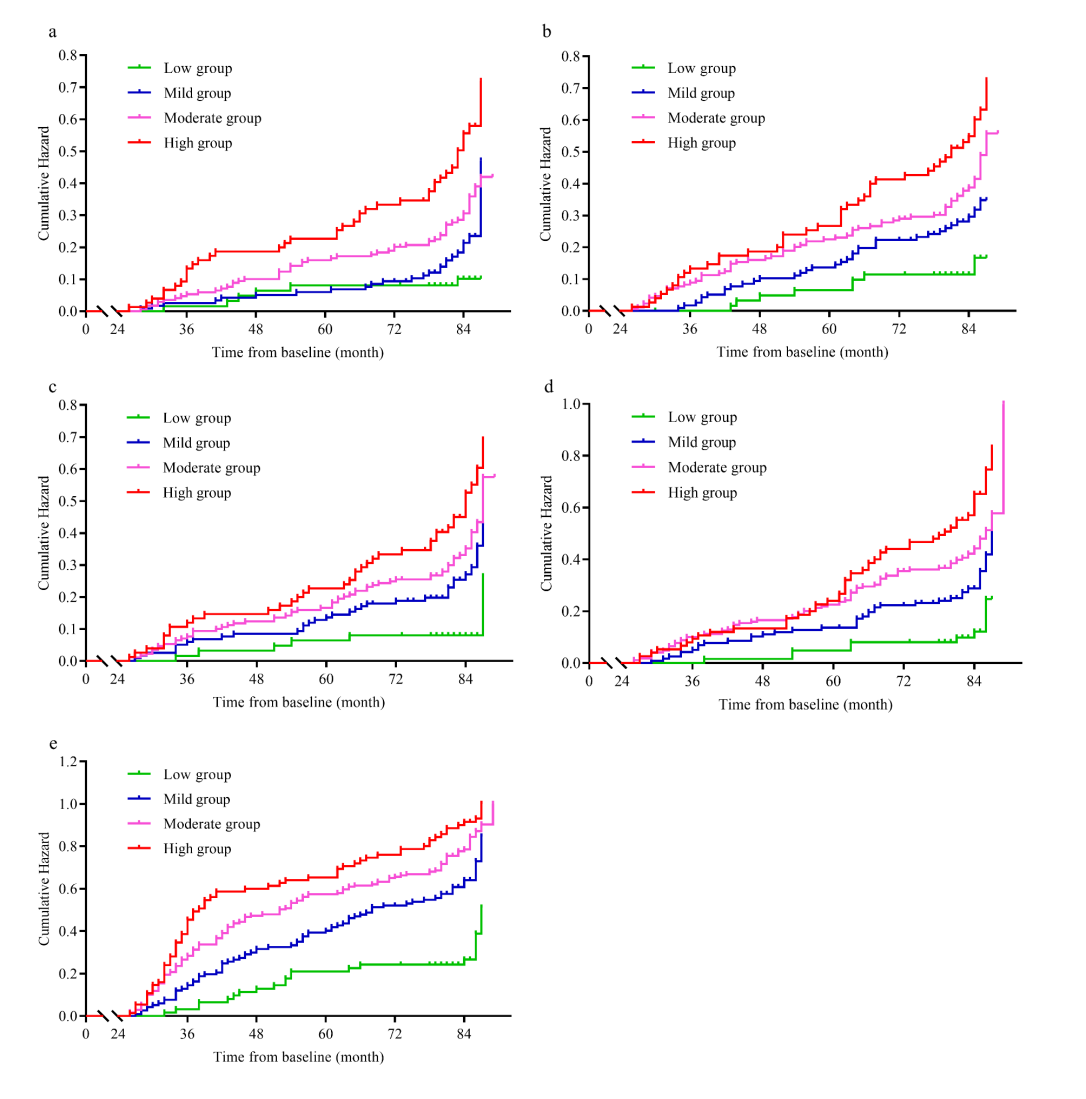
Differences in the cumulative hazard of CSVD progression among groups. (a) Differences in the cumulative hazard of new-incident WMH with Fazekas scale scores ≥ 2. (b) Differences in the cumulative hazard of new-incident lacunes. (c) Differences in the cumulative hazard of new-incident microbleeds. (d) Differences in the cumulative hazard of new-incident EPVS. (e) Differences in the cumulative hazard of the composite incidence of CSVD.

The new-incident WMH, lacunes, microbleeds, and EPVS in the low group were significantly lower than those in the mild, moderate and high groups (*P* < 0.05 for all, Table 2). The cumulative risks of new-incident WMH, lacunes, microbleeds and an EPVS were significantly higher in the mild, moderate and high groups compared with the low group after adjustment for confounders, with details shown in Fig. 3; Table 3 (all *P*_adjusted_ < 0.05). Similar results for the composite incidence of CSVD were also found (all *P*_adjusted_ < 0.05, Fig. 3; Table 3). The new-incident CSVD load in the mild, moderate and high groups was significantly greater than in the low group (all *P* < 0.001).

**Table 2:** Outcomes occurrences total and per year

	Total			Low group			Mild group			Moderate group			High group			*P* value
	Case (*n*)	% per year		Case (*n*)	% per year		Case (*n*)	% per year		Case (*n*)	% per year		Case(*n*)	% per year		
New-incident Fazekas scale ≥ 2	131	4.42		6	1.36		27	3.30^*^		56	4.73^*^		42	8.00*†‡		< 0.001
New-incident lacunes	159	5.37		8	1.84		35	4.27^*^		71	6.00^*†^		45	8.57*†‡		< 0.001
New-incident microbleeds	148	5.00		6	1.39		33	4.03^*^		67	5.66^*†^		42	8.00*†‡		< 0.001
New-incident EPVS	179	6.05		9	2.07		40	4.89^*^		80	6.76^*†^		50	9.53*†‡		< 0.001

EPVS indicates enlarged perivascular space. *P < 0.05, compared with the low group; †*P* < 0.05, compared with the mild group; ‡*P* < 0.05, compared with the moderate group

**Table 3:** Cumulative hazards of outcomes in the mild moderate, severe moderate, and high groups compared with low group

	HR	95% CI	*P* value
New-incident Fazekas scale ≥ 2			
Low group	Ref.		
Mild group	2.469	1.019 to 5.980	0.045
Moderate group	3.724	1.604 to 8.645	0.002
High group	7.393	3.141 to 17.399	< 0.001
New-incident lacunes			
Low group	Ref.		
Mild group	2.503	1.161 to 5.397	0.019
Moderate group	3.743	1.802 to 7.775	< 0.001
High group	5.852	2.757 to 12.421	< 0.001
New-incident microbleeds			
Low group	Ref.		
Mild group	3.325	1.393 to 7.938	0.007
Moderate group	4.664	2.022 to 10.756	< 0.001
High group	6.995	2.972 to 16.465	< 0.001
New-incident EPVS			
Low group	Ref.		
Mild group	2.699	1.309 to 5.564	0.007
Moderate group	4.008	2.011 to 7.990	< 0.001
High group	6.401	3.145 to 13.027	< 0.001
Composites of CSVD			
Low group	Ref.		
Mild group	2.893	1.751 to 4.780	< 0.001
Moderate group	4.486	2.775 to 7.253	< 0.001
High group	6.609	3.973 to 10.994	< 0.001

HR indicates hazard ratio; CI, confidence interval; EPVS, enlarged perivascular space; CSVD, cerebral small vessel disease

### Progression of CSVD with each 1-SD increment in salt intake

In this study, we also assessed the risk of progression of CSVD with each one-SD increment in salt intake. We found that the risk of new-incident WMHs was increased 74%, while the risk of new-incident lacunes, microbleeds and an EPVS increased 58%, 59% and 53%, respectively, and the composite incidence of CSVD increased 58% when salt intake increased by 1-SD across the follow-up period after adjustment for confounders (all *P*_adjusted_ <0.001, Table 4).

**Table 4:** Cumulative hazards of outcomes with each one-SD increment in Salt intake during the follow-up period

	HR	95% CI	*P* value
New-incident Fazekas scale ≥ 2	1.743	1.449 to 2.096	< 0.001
New-incident lacunes	1.578	1.341 to 1.857	< 0.001
New-incident microbleeds	1.590	1.342 to 1.883	< 0.001
New-incident EPVS	1.526	1.314 to 1.773	< 0.001
Composed incidence of CSVD	1.580	1.413 to 1.776	< 0.001

HR indicates hazard ratio; CI, confidence interval; EPVS, enlarged perivascular space; CSVD, cerebral small vessel disease

### Stroke incidence

**Fig. 4:**
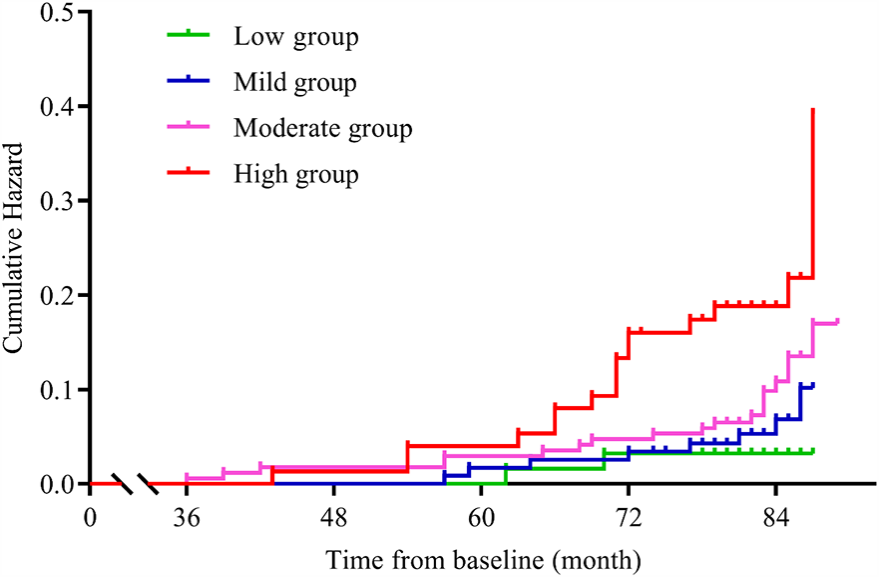
Differences in the cumulative hazard of stroke among groups across the duration of follow-up

In addition, we assessed the risk of stroke incidence in this study. There were 46 (1.7% per year) participants who suffered strokes. Among them, 2 (0.5% per year) were in the low group, 8 (1.0% per year) were in the mild group, 19 (1.6% per year) were in the moderate group and 17 (3.2% per year) were in the high group. The cumulative HRs were 2.16 (95% CI: 0.46–10.18, *P*_adjusted_ = 0.330) for the mild group, 3.47 (95% CI: 0.81–14.89, *P*_adjusted_ = 0.095) for the moderate group and 7.56 (95% CI: 1.75–32.72, *P*_adjusted_ =0.007) for the high group after adjustment for confounders (Fig. 4). The risk of stroke was increased 74% (95% CI: 1.27–2.38, *P*_adjusted_ = 0.001) with each 1-SD increment in salt intake after adjustment for confounders.

## Discussion

The main finding of this prospective cohort study was that the risk for progression of CSVD including WMLs, lacunes, microbleeds and an EPVS was significantly greater in the higher salt-intake participants than in lower salt-intake participants. There was a significant and independent increased risk for progression of CSVD with each 1-SD increment in salt intake even after adjustment for confounders including smoking, blood pressure, blood lipids, fasting plasma glucose, and baseline WMH volume. In addition, we found that the risk of stroke incidence was increased with the increment of salt intake.

Although salt is one of essential nutrients for the human body, excessive salt intake is believed to be an unhealthy dietary pattern. The links between excessive salt intake and hypertension, atherosclerosis and stroke have been established [15, 24, 25], but there are few studies on the association between excessive salt intake and CSVD. Heye [26] and Makin [10] recruited lacunar and cortical stroke patients to investigate the correlation between salt intake and CSVD. Their results showed that salt intake was positively associated with increased WMHs [26]. The risks of lacunar stroke, lacunes, microbleeds, severe WMHs and worse CSVD scores were increased approximately 1.9-, 2.1-, 3.4-, 2.5- and 2.2-fold, respectively, in patients with excessive salt intake compared to patients with normal intake [10]. However, these studies were cross-sectional designs with a small sample size and cannot explain the causal relationship between salt intake and the progression of CSVD due to the inherent characteristics of cross-sectional studies. Furthermore, in these studies, participants were stroke patients and the salt intake was estimated using a questionnaire. The questionnaire may partially but inaccurately reflect the actual daily salt intake.

In this study, we used a prospective cohort design, which avoids the defects of cross-sectional studies, to clarify the causal link between excessive salt intake and the progression of CSVD. Our results demonstrated that the risks of WML progression and new-incident lacunes, microbleeds and an EPVS in participants with higher salt intake were significantly higher than in participants with lower salt intake. Meanwhile, we found that the risk of CSVD progression including WMLs, lacunes, microbleeds and an EPVS was significantly increased with each 1-SD increment in salt intake. Excessive salt intake was positively and independently associated with a higher risk of CSVD progression even after adjustment for baseline WMH volume as well as blood pressure and histories of hypertension and antihypertensive medication. Our results indicate that excessive salt intake may be an important contributor to CSVD. The mechanism underlying excessive salt intake on CSVD could involve changes in gene and protein expression [27], inflammatory processes [28, 29] and salt sensitivity [30].

In addition, 24-hour urine collection for seven consecutive days was used to estimate the daily salt intake in this study. This method is regarded as the current gold-standard measurement of dietary salt intake and can maximize the accuracy of daily salt intake estimation. Indeed, the accuracy of this method is actually affected by some factors such as season, sweating and age [16]. To eliminate possible bias, urine collection in the present study was performed in the Spring or Autumn to minimize the impact of sweating. We also used urine creatinine excretion to correct and elevate salt intake estimation, because almost all creatinine produced in the body every day is excreted from urine [31].

We also observed the association between risk of stroke and salt intake. Similar to the progression of CSVD, our results indicated that excessive salt intake was closely associated with a high risk for stroke and agreed with the findings of previous studies [6, 32].

Studies have shown that there is a J-shaped association of dietary salt intake with mortality and cardiovascular events [33, 34]. In this study, we found that the new-incident CSVD was positively correlated with salt intake and the J-shape curve was less obvious. The small sample might be the major cause to induce the bias. The minimum of salt intake of participant was 4.4 g/day in this study. Dietary sodium intake over a certain physiological minimum level has been demonstrated to be associated with cardiovascular diseases such as atrial fibrillation [35].

The major strength of this study was its prospective design and long-term follow-up. In addition, we used the gold-standard method to estimate the salt intake. However, there are some limitations of our study which should also be addressed. First, the changes of salt intake style were determined by spot urine sample. This may have induce a bias in the results if the participants’ dietary patterns changed during the long-term follow-up. Second, we did not assess the entire dietary patterns including total energy intake and potassium intake in the study. Excessive salt intake has been demonstrated to be closely associated with total energy and potassium intake [36], which may affect the progression of CSVD. Third, the participants in this study were mainly Han Chinese recruited from the Shandong area of China. Evidence has shown with some debate that ethnic origin and race may influence the progression of CSVD [37, 38].

## Conclusion

In conclusion, our results demonstrate that excessive salt intake aggravates the progression of CSVD in older adults. Reducing salt intake will be of benefit for healthy management of older individuals. However, further studies including multiethnic individuals and energy and potassium intake are needed. The mechanism underlying excessive salt intake on the progression of CSVD should also be thoroughly elucidated in the future.

## Data Availability

The dataset used and/or analysed during the current study is available from the corresponding author on reasonable request.
